# A moth pheromone brewery: production of (*Z*)-11-hexadecenol by heterologous co-expression of two biosynthetic genes from a noctuid moth in a yeast cell factory

**DOI:** 10.1186/1475-2859-12-125

**Published:** 2013-12-13

**Authors:** Åsa K Hagström, Hong-Lei Wang, Marjorie A Liénard, Jean-Marc Lassance, Tomas Johansson, Christer Löfstedt

**Affiliations:** 1 Pheromone Group, Department of Biology, Lund University, Lund, Sweden; 2 Hartl Laboratory, Department of Organismic and Evolutionary Biology, Harvard University, Cambridge, MA, USA; 3 Hoekstra Laboratory, Department of Organismic and Evolutionary Biology, Harvard University, Cambridge, MA, USA; 4 MEMEG, Department of Biology, Lund University, Lund, Sweden

## Abstract

**Background:**

Moths (Lepidoptera) are highly dependent on chemical communication to find a mate. Compared to conventional unselective insecticides, synthetic pheromones have successfully served to lure male moths as a specific and environmentally friendly way to control important pest species. However, the chemical synthesis and purification of the sex pheromone components in large amounts is a difficult and costly task. The repertoire of enzymes involved in moth pheromone biosynthesis *in insecta* can be seen as a library of specific catalysts that can be used to facilitate the synthesis of a particular chemical component. In this study, we present a novel approach to effectively aid in the preparation of semi-synthetic pheromone components using an engineered vector co-expressing two key biosynthetic enzymes in a simple yeast cell factory.

**Results:**

We first identified and functionally characterized a ∆11 Fatty-Acyl Desaturase and a Fatty-Acyl Reductase from the Turnip moth, *Agrotis segetum*. The ∆11-desaturase produced predominantly Z11-16:acyl, a common pheromone component precursor, from the abundant yeast palmitic acid and the FAR transformed a series of saturated and unsaturated fatty acids into their corresponding alcohols which may serve as pheromone components in many moth species. Secondly, when we co-expressed the genes in the Brewer’s yeast *Saccharomyces cerevisiae*, a set of long-chain fatty acids and alcohols that are not naturally occurring in yeast were produced from inherent yeast fatty acids, and the presence of (*Z*)-11-hexadecenol (Z11-16:OH), demonstrated that both heterologous enzymes were active in concert. A 100 ml batch yeast culture produced on average 19.5 μg Z11-16:OH. Finally, we demonstrated that oxidized extracts from the yeast cells containing (*Z*)-11-hexadecenal and other aldehyde pheromone compounds elicited specific electrophysiological activity from male antennae of the Tobacco budworm, *Heliothis virescens*, supporting the idea that genes from different species can be used as a molecular toolbox to produce pheromone components or pheromone component precursors of potential use for control of a variety of moths.

**Conclusions:**

This study is a first proof-of-principle that it is possible to “brew” biologically active moth pheromone components through in vitro co-expression of pheromone biosynthetic enzymes, without having to provide supplementary precursors. Substrates present in the yeast alone appear to be sufficient.

## Background

Moths and butterflies (Lepidoptera) form an order of insects that includes more than 174,000 described species [1]. Moths are among the most damaging pests of food and fiber crops, and are capable of adapting fast and evolving resistance to insecticides [2]. Conventional insecticides are not discriminative between pest and other non-target insects and can in many cases be harmful to other organisms, including humans, and detrimental to plants that are dependent on beneficial insects for pollination [3]. Species-specific control systems such as synthetic pheromones for pest insects have been developed to aid in maintaining a more sustainable agriculture.

Mate finding in moths typically involves species-specific female-produced pheromones. They usually consist of long–chain fatty acid derivatives (alcohols, acetates, aldehydes) released, either as a single-component pheromone or as multi-component blends in specific ratios [4,5]. The specificity of the pheromone systems thus makes it possible to develop environmentally friendly and moth-specific pest-management strategies. There are several possibilities for pheromone-based pest control [6]. So far, mating disruption has been the most successful method: by releasing synthetic pheromone into the crop field, the natural female-produced pheromone is masked or synthetic sources compete with the females. As a consequence males have difficulties to locate their potential partners. Other ways to use pheromones in pest control include mass-trapping with pheromone lures, or push-and-pull where the moths are repelled from the crop with one stimulus while simultaneously attracted to other areas with attractive lures. Pheromones can also be used for monitoring purposes. Doing so, they can help in rationalizing and decreasing the use of conventional pesticides [7].

For large-scale commercial production, the pheromone components are traditionally synthesized chemically, which often requires harmful reactants and may generate chemical waste [8]. The starting material when synthesizing moth pheromone components is typically saturated fatty alcohols that can be produced from natural sources by hydrolysis of wax esters from sperm oil, reduction of wax esters with sodium, or by hydrogenation of natural raw materials. Alcohols can also be produced from petrochemical feedstocks, for example using the Ziegler alcohol processes, the oxo process (hydroformylation), hydrogenation of fatty acids produced by oxidation of paraffinic hydrocarbons, or the Bashkirov Oxidation. The production of unsaturated fatty alcohols requires selective hydrogenation using various metal oxides as catalysts [9]. To synthesize the acetate and aldehyde pheromone compounds, further chemical processing of the fatty alcohols is necessary. Typically, the alcohols can be transformed to their corresponding aldehydes using the oxidant pyridinium chlorochromate (PCC), and the corresponding acetates are formed by acetylchloride-based acetylation [10].

Biosynthetically, moth sex pheromones are produced *de novo* from fatty-acyl (FA) precursors by a series of enzymatic steps involving desaturation, chain elongation, and chain shortening that produce intermediates with specific chain lengths, double bond position(s), and geometries. The structures are then completed by the adjustment of the functional group by reduction, oxidation, or transesterification to provide alcohols, aldehydes, or acetates, respectively, that serve as the actual volatile pheromone components [4,11]. The evidence available so far suggests that all of these transformations are carried out in the female pheromone gland, in which a set of specialized enzymes has evolved to fulfill these particular biosynthetic functions [12]. The most thoroughly studied groups of pheromone biosynthetic enzymes are the Fatty-Acyl Desaturases (FAD) and the pheromone gland Fatty-Acyl Reductases (pgFAR). The former group includes a wide repertoire of FADs capable of performing both highly specific desaturations at various positions along the carbon backbone, such as ∆9, ∆11, and ∆14, in either *Z* or *E* configuration (or both), as well as multi-substrate and multi-functional enzymes that can introduce multiple unsaturations [13-16]. The pgFARs consist of both highly stereo/regiospecific members [17-19] as well as a group of promiscuous enzymes [20,21].

The moth pheromone biosynthetic enzymes are a potential goldmine for biochemical applications. Since moths are able to synthesize a plethora of various chemicals via a simple enzymatic machinery, we envisioned that this system could be applied to “brew” a pheromone or pheromone precursors in yeast. In particular, the Brewer’s yeast, *Saccharomyces cerevisiae* naturally produces a multitude of fatty acids [22], and these are ideal substrates for both pheromone biosynthetic FADs [13-15,23] and pgFARs [17,20,21].

**Figure 1:**
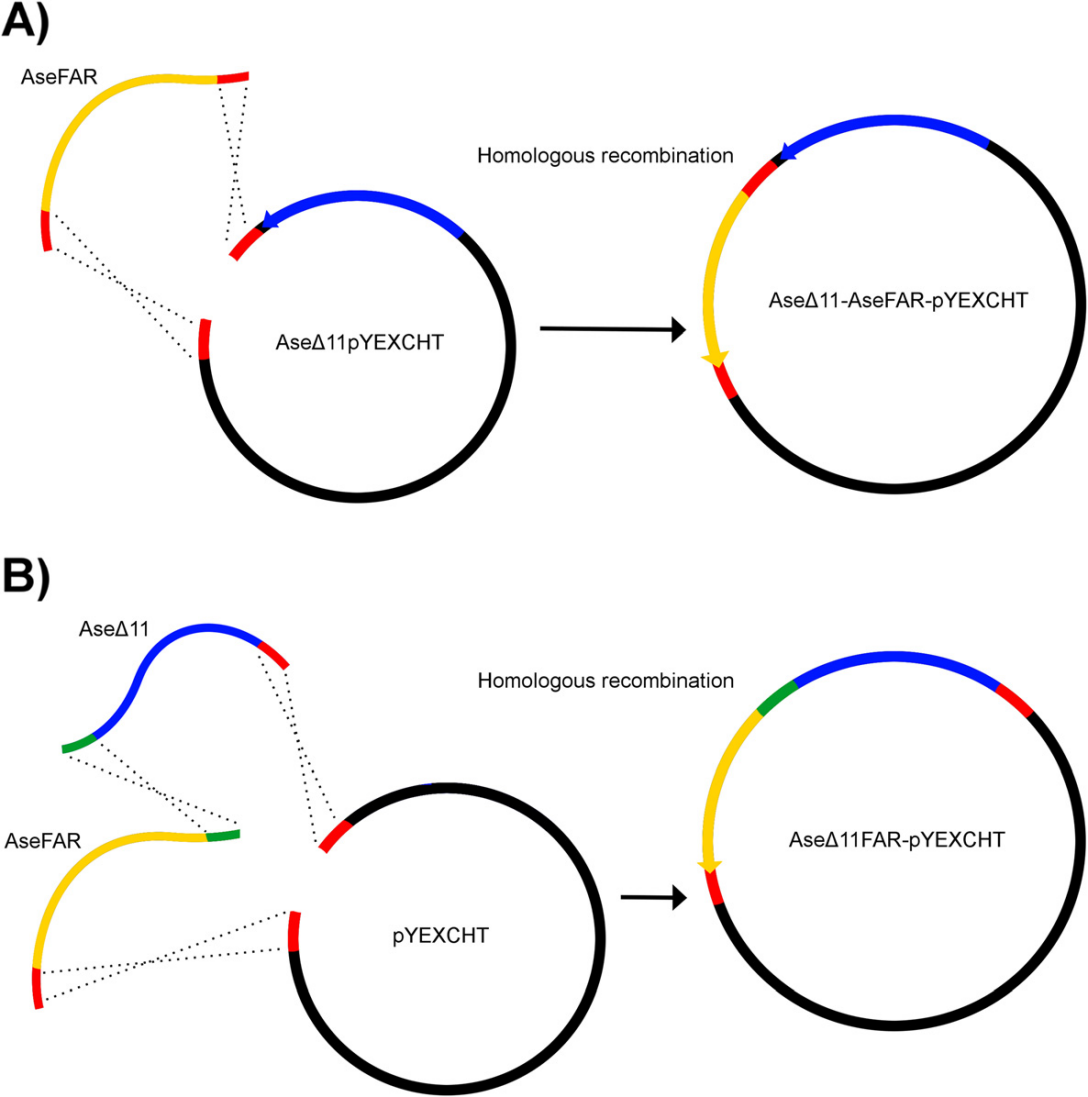
***Constructs for co-expression of AseΔ11 and AseFAR in yeast*. A)** represents the generation of the construct Ase∆11/*CUP1p*-AseFAR/*GAL1p*-pYEXCHT by homologous recombination through co-transformation in *Saccharomyces cerevisiae* of the AseFAR (yellow), which is flanked with short sequences (red) homologous to the PvuII-linearized pYEXCHT already carrying Ase∆11 (blue). **B)** represents how the two ORFs were fused into a single ORF by *S. cerevisiae* homologous recombination, generating the construct Ase∆11-FAR/*CUP1p*-pYEXCHT. The ORFs are flanked with a short sequence (red) homologous to the XhoI-linearized pYEXCHT on one side, as well as with a short homologous sequence interspacing the two ORFs (green) for the purpose to aid fusion into a chimeric ORF.

In this article, we report on the functional characterization of a FAD with Δ11-activity and a broad-acting pgFAR isolated from the Turnip moth, *Agrotis segetum* (Figure 1A-B) and investigate their heterologous co-expression by constructing a two-step pheromone biosynthetic pathway in yeast with the aim of producing (*Z*)-11-hexadecenol (Z11-16:OH, [24]), a common moth pheromone component, de novo. Our results constitute a proof of concept that it is indeed feasible to produce one of the most frequently used moth sex pheromone components in this way. With an increasing repertoire of characterized insect FADs and FARs at hand, this method has the potential to assist in the semi-synthetic production of various species-specific moth sex pheromone components.

## Results and discussion

### AseΔ11 and AseFAR identification, phylogenetics, and functional assay

**Figure 2:**
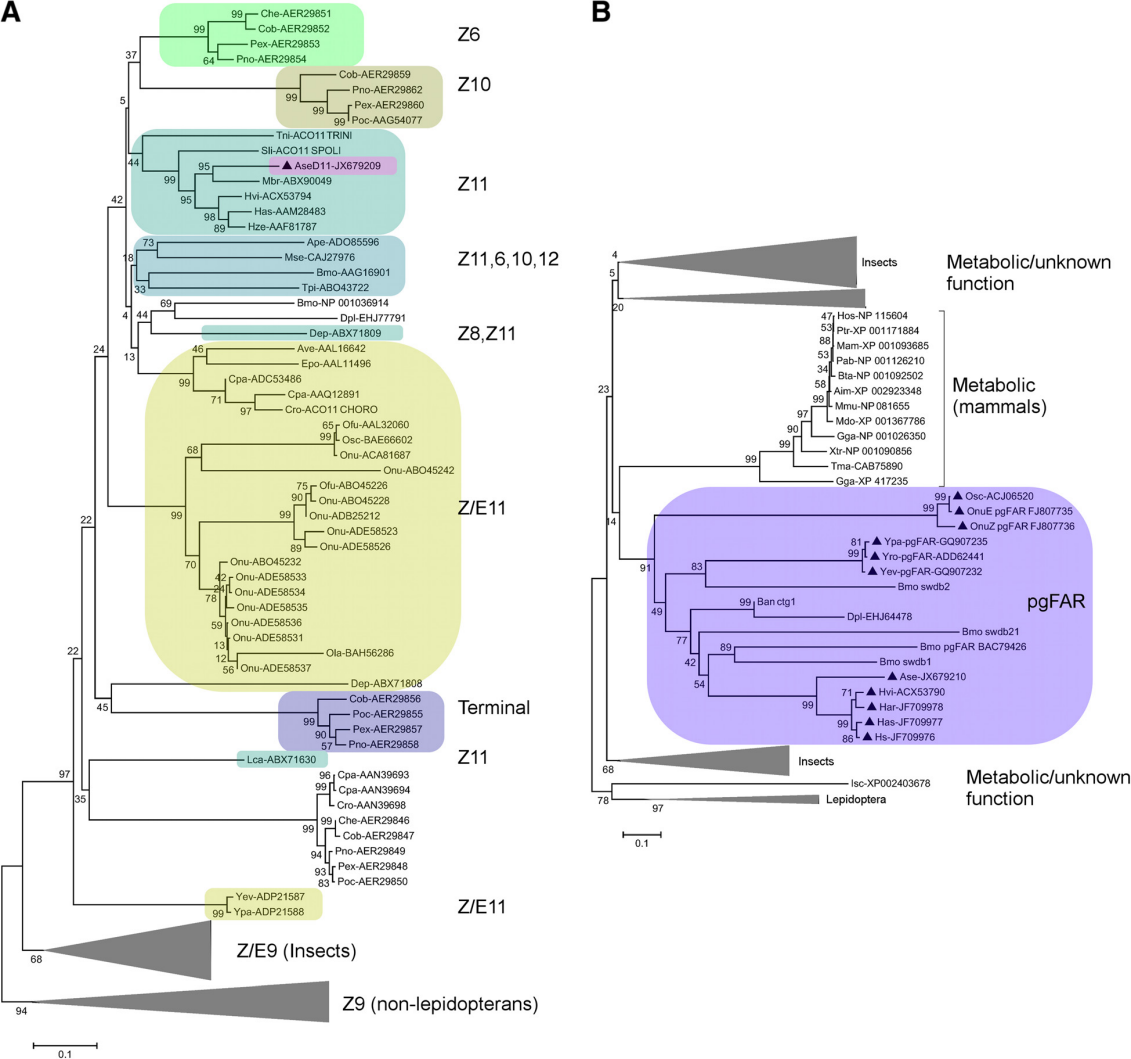
***Phylogenetic analysis of AseΔ11 and AseFAR*. A)** Shows a Fatty-Acyl Desaturase (FAD) phylogenetic tree, in which Ase∆11 (pink) clusters with moth ∆11-desaturases in a subgroup of ∆11 noctuid homologs (grey green). **B)** Shows a Fatty-Acyl Reductase (FAR) phylogenetic tree, where the AseFAR clusters in a well-supported group (purple) of lepidopteran pgFARs (marked with triangles). Species abbreviations can be found in Additional file 5.

DNA sequencing of a pheromone gland EST clone (Acc. No ES583599, [25]) yielded a full-length AseΔ11 ORF of 1011 nt corresponding to a 336 aa protein, which clustered within a group of noctuid Δ11-desaturases in our phylogenetic analysis (Figure 2). The reconstructed phylogeny forms a catalogue of an enormous diversity of moth FADs, which are able to perform enzymatic reaction activities not known from other species. The ∆11-subfamily contains rapidly evolving genes with various activities, including not only Δ11 but for instance also Δ10, Δ6 and bifunctional enzymes [26-28]. Other non-lepidopteran, arthropod desaturases and vertebrate desaturases function as common metabolic Δ9-desaturases.

**Figure 3:**
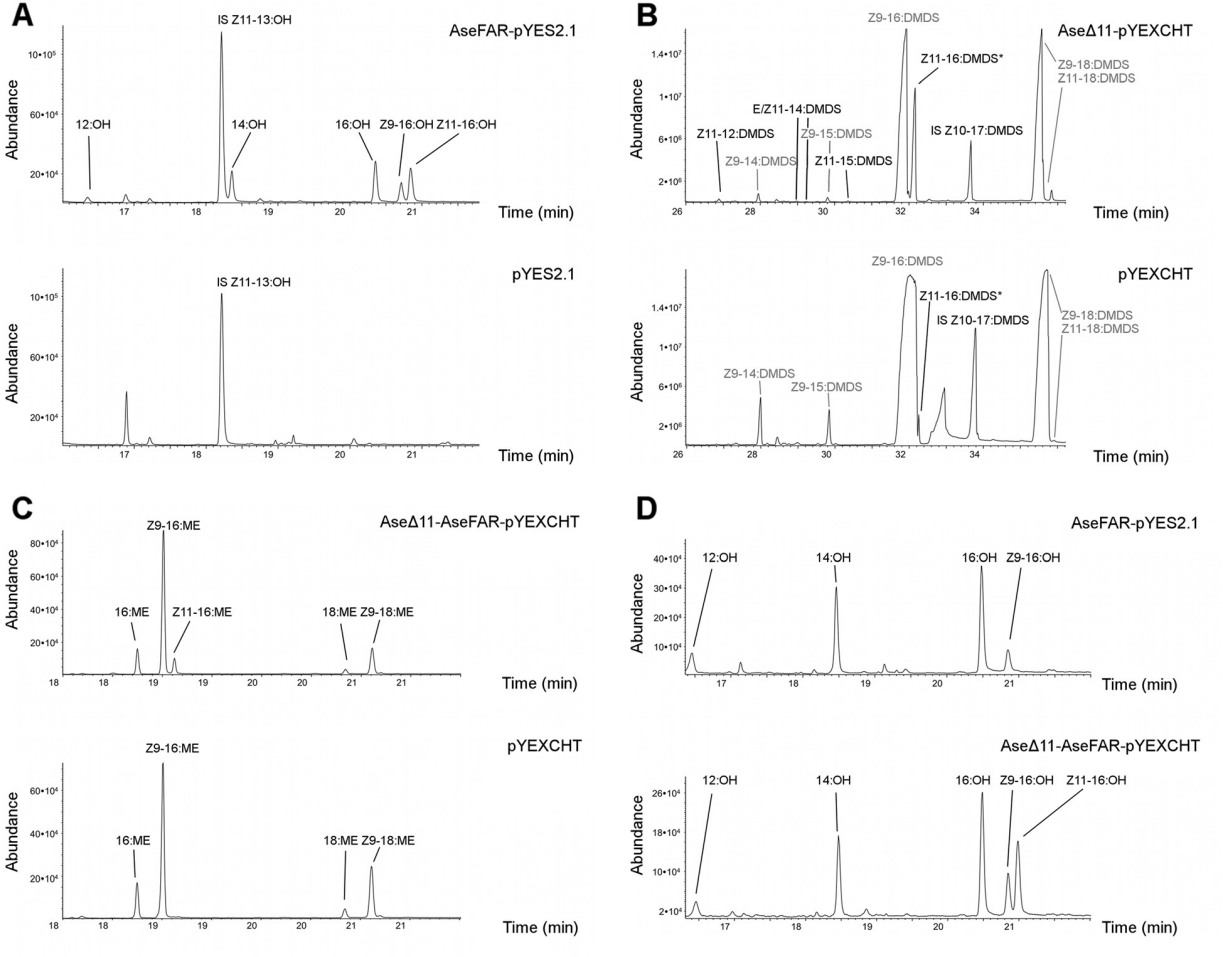
***Expression and co-expression of AseΔ11 and AseFAR in *S. cerevisiae. A)** Alcohol products from AseFAR expression (top) supplemented with Z11-16:ME, compared to vector only negative control (bottom). AseFAR produces the corresponding Z11-16:OH, as well as 12:OH, 14:OH, 16:OH, and Z9-16:OH, as a result from conversion of natural FA substrates present in yeast. None of these alcohols are found in the negative control. **B)** DMDS derivatization of Ase∆11-expressing yeast subjected to base methanolysis (top) compared to an empty vector control (bottom). The Ase∆11 chromatogram shows peaks corresponding to ∆11-12:DMDS, Z11-14:DMDS, E11-14:DMDS, and Z11-15:DMDS, which are not present in the negative control. Additionally, there is a substantially higher peak in AseΔ11 corresponding to Z11-16:DMDS (also see additional file 1). **C)** Base methanolysed yeast extract from Ase∆11/*CUP1p*-AseFAR/*GAL1p*-pYEXCHT (top panel) as compared to a negative control (bottom panel) showing the presence of Z11-16:ME in the former but not the latter. **D)** Alcohol extracts from AseFAR-pYES2.1 (top panel) compared with Ase∆11/*CUP1p*-AseFAR/*GAL1p*-pYEXCHT (bottom panel). Both samples contain 12:OH, 14:OH, 16:OH, and Z9-16:OH, but only the latter contains Z11-16:OH.

When *AseΔ11* was heterologously expressed in *S. cerevisiae*, the GC-MS analysis of base methanolysed cell extracts revealed the presence of an abundant peak corresponding to a hexadecenoic methyl ester with the characteristic ions *m/z* 268 and 236. The DMDS (dimethyl disulfide) derivative of this ester displayed the characteristic ions at *m/z* 245, 117 and  362, thus confirming a Δ11-double bond position. Further comparison of retention times with the reference compounds (*E*)-11-hexadecenoic acid methyl ester (E11-16:ME) and (*Z*)-11-hexadecenoic acid methyl ester (Z11-16:ME) confirmed a ¨*Z*¨ configuration of the double bond (Figure 3B and Additional file 1). AseΔ11-pYEXCHT produced significantly large amounts of (*Z*)-11-hexadecenoic acid (Z11-16:COOH), whereas the negative control (empty vector) produced much less of Z11-16:COOH, likely resulting from endogenous elongation of (*Z*)-9-tetradecenoic acid (Z9-14:COOH). In addition to Z11-16:COOH, the AseΔ11-pYEXCHT extract also contained (Δ)-11-dodecenoic acid (Δ11-12:COOH), (*Z*)-11-tetradecenoic acid (Z11-14:COOH)*,* (*E*)-11-tetradecenoic acid (E11-14:COOH) and (*Z*)-11-pentadecenoic acid (Z11-15:COOH), as confirmed by the DMDS adducts of the methanolysis products. None of these acids were present in the negative control (Figure 3B). This Δ11-desaturase activity profile is a common pattern found in many moth species. Yet it is interesting that the Δ11-desaturase found in *A. segetum* shows no activity on 18:COOH but instead activities shifted towards C12-C16 acids. This distinguishes AseΔ11 from other identified and functionally characterized Δ11-desaturases that phylogenetically clustered into the same clade (Figure 2): its homolog found in *Helicoverpa zea* which is 16:COOH-specific [16], and possibly also from the homologs in *Trichoplusia ni*[27,28] and *Spodoptera littoralis *[29] that act on C14-C18 acids, as well as from an inactive copy from *Mamestra brassicae *[30]. This again exemplifies the biochemical diversity present amongst a small fraction of known moth pheromone biosynthetic Δ11-FADs, as compared to the conserved specificity and activity in Δ9-desaturase homologs, which are mostly metabolic.

The 5’-RACE PCR of AseFAR from an *A. segetum* female pheromone gland cDNA combined with the available EST library contig sequence information led to obtain the full ORF corresponding to 1377 nt that translated into a 458 aa protein. AseFAR clustered into and confirmed the previously well-supported Lepidoptera-specific pgFAR group [18,20], including a subgroup of other broad-acting noctuid pgFARs (Figure 2). Heterologous expression of AseFAR in *S. cerevisiae* supplemented with Z11-16:ME produced the corresponding Z11-16:OH, and when supplemented with Z11-16:Me, Z9-14:ME and Z9-16:ME, additionally produced the corresponding Z9-14:OH, Z9-16:OH (Figure 3A and Additional file 2), of which neither were present in the negative control. Yeast extracts also contained saturated alcohols (10:OH, 12:OH, 14:OH and 16:OH), which were produced by the AseFAR from the natural yeast fatty acid pool. This promiscuous activity pattern was previously found in pgFARs from four other noctuid moths [21], indicating a relatively broad substrate selectivity of the noctuid pgFARs.

### Heterologous co-expression of AseFAR and AseΔ11

A small-scale assay of AseΔ11/*CUP1p*-AseFAR/*GAL1p*-pYEXCHT successfully produced Z11-16:COOH as detected by its methanolysis product when the *CUP1* promoter (*CUP1p*) was induced (Figure 3C). When the *GAL1* promoter (*GAL1p*) was subsequently induced the corresponding alcohol Z11-16:OH was produced (Figure 3D). This showed that both enzymes are functional in the multi-gene expression construct. In contrast, Z11-16:OH was not produced intrinsically by the yeast (Figure 3A), or by the yeast carrying the ‘reductase-only’ construct AseFAR-pYES2.1 (Figure 3D).

The ORF fusion was tested based on the concept of substrate channeling, *i.e.* in nature enzymes that are performing subsequent steps in a pathway are often located in close proximity so that the effective concentration of pathway intermediates near their recipient active site is increased and hence catalytic efficiency is boosted [31]. There was however no particular difference in the production of Z11-16:OH between the two assays, of which one construct had both genes co-expressed as two separate ORFs under the control of individual promoters, whereas the other construct had the two genes fused into one chimeric single ORF AseΔ11-FAR/*CUP1p*-pYEXCHT under the control of a single promoter (Additional file 3). We therefore continued our investigation with the AseΔ11/*CUP1p*-AseFAR/*GAL1p*-pYEXCHT construct in which each ORF is linked to a distinct inducible-promoter, since it enabled an independent control of both genes. Since the ORF fusion did not increase the production of Z11-16:OH, we suggest that the enzymes are co-localized in the yeast cells independently of whether they are expressed separately or synthetically fused together. This is in agreement with the localization into the endoplasmic reticulum of both the FAD [32-34] and the pgFARs [35]. Further manipulation to either increase the production of Z11-16:OH alone or obtain other double bond and chain-length specific alcohols could be achieved by either choosing a different host strain that produces a certain ratio of saturated fatty acids, or using more specific FAD and pgFAR genes. In the latter group however, there are so far not that many reductases found that are specific for a certain substrate. Another unexplored option to get high production of target compound would be to evolve improved candidates by manipulation of the related enzymes via directed evolution [36].

Microbial enzymes have been used for various industrial applications, either alone or for ‘hybrid processes’ of enzymatic and chemical reactions, improving the production of amino acids, sweeteners, oils, vitamins, chemicals, pharmaceutical intermediates, etc. [37]. Using *S. cerevisiae* as a cell-based factory for production of moth pheromone components or precursors is advantageous compared to other systems in many ways: yeast cultures grow fast and adapt well in controlled environments; yeast is considered as a GRAS organism; and in addition, there exist a substantial number of tools for their genetic engineering and manipulation. Various isoprenoids and long-chain polyunsaturated fatty acids of commercial value can nowadays be produced in yeast by heterologous expression of enzymes from other organisms [38]. Our lab-scale assay of a 100 ml AseΔ11/*Cup1p*-AseFAR/*GAL1p*-pYEXCHT incubation produced on average 19.5 μg Z11-16:OH (±1.6), 49.7 μg 14:OH (SEM ±3.06), 2.9 μg Z9-14:OH (±0.25), 67.8 μg 16:OH (±7.2), and 26.3 μg Z9-16:OH (±2.4). This provides a proof-of-concept that partial or complete pheromone biosynthetic pathways can be reconstructed in yeast. The produced yeast extracts could be either used as moth pheromones directly (as when yeast extract showed to attract *Bombyx mori* males when expressing the pgFAR [17]) or chemically processed to get desired derivates.

### Gas chromatography with electroantennographic detection (GC-EAD) activities of the oxidized yeast products

**Figure 4:**
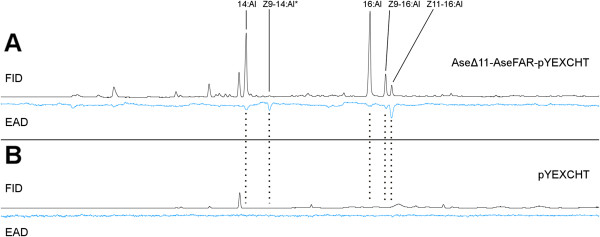
**Gas chromatography of oxidized yeast extracts. (A)** When stimulated with the oxidized extract from Ase∆11/CUP1p-AseFAR/GAL1p-pYEXCHT the *H. virescens* male antenna responds to the pheromone components Z9-14:Al and Z11-16:Al, as well as to 14:Al, Z9-16:Al and to a lesser extent to 16:Al. **(B)** In the negative control there are no aldehydes and no antennal responses to the oxidized yeast extract at corresponding retention times (dashed lines), or at any other elution time. Flame ionization detector (FID) response is marked in black, while electroantennographic detector (EAD) response using a male *H. virescens* antenna is marked in blue. Repeated experiments produced the same EAD response pattern.

To validate the biological activity of the oxidized yeast extract and to support the idea that genes from one species can be used to produce components for other species as well, the Tobacco budworm, *Heliothis virescens* was selected as an experimental insect. There is a high structural similarity between the sex pheromone of this species, which mainly consists of (*Z*)-11-hexadecenal (Z11-16:Al) and (*Z*)-9-tetradecenal (Z9-14:Al) with traces of other aldehydes (Additional file 4), and the oxidized yeast extract, *i.e*., the aldehydes resulting from oxidation of the alcohols (Table 1). When *H. virescens* male antennae were subjected to a synthetically oxidized yeast extract resulting from the co-expression of AseΔ11 and AseFAR, there were strong responses to the major pheromone component Z11-16:Al, and the minor pheromone component Z9-14:Al (produced in minor amounts), as well as to 14:Al, 16:Al, and Z9-16:Al (Figure 4A). This was in agreement with our expectations and proved the electrophysiological activity of the oxidized yeast co-expression products, at the same time demonstrating the absence of side-products with potentially interfering electrophysiological activity. The oxidized extract from the negative control did not elicit any antennal response (Figure 4B).

**Table 1:** **Aldehyde composition of the converted yeast extract in relative amounts (percentage) from AseFAR-∆11-pYEXCHT compared to the proportions of aldehydes found in the gland of *H. virescens *** [50]

**Compound**	**AseFAR-∆11-pYEXCHT**	***Heliothis virescens***
14:Al	30	13
Z9-14:Al	2	18
16:Al	41	7
Z7-16:Al	0	1
Z9-16:Al	16	1
Z11-16:Al	11	60

Our results support the possibility to “brew” moth pheromones in yeast and potentially use the extract directly as a lure for moth trapping or even pest control via pheromone-mediated mating disruption (MD). The amount of pheromone needed for pest-control highly depends on the target moth species. For example, the optimal dose of synthetic pheromone on a rubber septum dispenser for attraction of the Spruce seed moth (*Cydia strobilella*) is as low as 0.3 μg, while an optimal lure for the apple leaf-miner (*Phyllonorycter ringoniella)* is 1 mg per lure [39]. The amounts of pheromone compounds produced in our small-scale yeast assay would in many cases suffice to produce baits for monitoring of selected moth pests. Larger scale production of pheromones for mating disruption could eventually be achieved using fermenters, after optimization of media and growing conditions, or even change of host strain.

Trapping of a particular species would require an optimized ratio of the yeast products that match the pheromone composition of that species. This may not be possible “in one pot” but may be achieved with a set-up of different gene combinations producing different product ratios and subsequently blending the yeast products to obtain specific final ratios required by target moth species. The possibility to construct “tailored” enzymes based on already characterized moth pheromone biosynthetic enzymes as templates can in addition be a tool to generate isoforms that display differences in substrate selectivity, which has been done with the pgFARs of *Ostrinia* spp. [19]. The ultimate “pheromone brewery” would preferably not need any substantial chemical post-processing, but neither the alcohol oxidase(s) nor the acetyltransferases that are responsible for the production of aldehydes and acetates from fatty alcohols have been identified and characterized yet. For application purposes this may not be a problem as long as the system can accept combinations of enzymes from another organism, *e.g.* in *Escherichia coli*, the fatty acid composition has been altered through co-expression of a plant acyl-acyl carrier protein desaturase and ferredoxin, which significantly increased the amount of Δ6-hexadecenoic acid [40]. Desaturases from microalgae have also been co-expressed with a moss Δ6-elongase or an algal Δ5-elongase to establish an acyl-CoA-dependent pathway of ω3-very long-chain polyunsaturated fatty acid production in yeast [41]. Another potential that lies in the co-expression system with moth pheromone biosynthetic enzymes is to express two desaturase types and produce specific polyunsaturated FA or alcohols. This would be similar to what has been done for instance in the yeast *Yarrowia lipolytica*, in which a Δ6- and a Δ12-desaturase were expressed simultaneously to increase the production of γ-linoleic acid [42]. Moths show an enormous variation in pheromones based on a common theme. This is the consequence of unique enzymes, of which certain classes remain undiscovered but the FADs and FARs that we have at hand already offer unique opportunities to “brew” a large set of pheromone precursors and fatty alcohols that may be chemically modified.

## Conclusions

Sex pheromones in moths form highly specific communication channels on which the insects are fully dependent in order to find a mate, a system that has successfully been exploited to develop pest-control methods based on synthetic pheromones. Previous studies have successfully identified and functionally characterized an increasing number of FADs and pgFARs involved in moth pheromone biosynthesis. The present study further adds to this diversity with the identification of two pheromone biosynthetic genes from *A. segetum* and their co-expression in yeast: a typical FAD with ∆11 activity on C12-16 FA substrates and a promiscuous pgFAR active on both saturated and unsaturated substrates with chain-lengths varying from C10 to C16. After co-expressing both enzymes in yeast we were able to recover a mixture of fatty alcohols in particular the Z11-16:OH, and further demonstrate the biological activity of the chemically derived aldehydes on male moth antennae. Not only does our approach provide a study system in which it is possible to focus on two important subsequent steps in moth pheromone biosynthesis, but it also provides a platform for developing pheromone and precursor production in a natural and environmentally friendly way. The transcriptomics era provides more and more data on potential FADs, pgFARs and other candidate genes, and will most likely lead to the identification of the remaining pheromone biosynthetic key enzymes [12]. By making use of the enzyme sets characterized from moth species so far, we already have a molecular toolbox, which can aid in pheromone production and insect control and reduce the unwanted environmental consequences of conventional insecticides and synthetic pheromone production.

## Methods

### Insects

*A. segetum* originated from our laboratory-reared strain maintained on artificial soybean diet. *H. virescens* originated from a mixed population and were reared on the same diet as *A. segetum*. Male and female pupae were separated and kept in rearing chambers at 22 ± 1°C in a 19-h:5-h light:dark photoperiod.

### Total RNA extraction and cDNA preparation

Female pheromone glands were dissected from 1–4 day old individuals during early scotophase, and RNA was extracted using the RNeasy Mini Kit (Qiagen) according to the manufacturer’s instructions. First-strand cDNA was prepared from 1 μg total RNA using the Stratagene cDNA Synthesis Kit and an oligo-dT primer (Stratagene, AH Diagnostics, Skärholmen, Sweden). For pyrosequencing, cDNA was prepared from 1 μg of pooled total RNA. The cDNA was pyrosequenced using a Roche 454 GS-FLX system and the sequence reads assembled into contigs. Detailed methods and results of the EST library will be presented elsewhere.

### Phylogenetic analysis

The full-length AseΔ11 aa sequence was analyzed by protein BLAST in GenBank non-redundant (nr) protein database [43] (NCBI) by four different approaches: 1) the first 100 sequences similar to AseΔ11 by default settings were downloaded, 2) Lepidoptera and Drosophila sequences were excluded from the query and the first 20 homologous sequences were downloaded, 3) Insect sequences were excluded and the first 20 homologous sequences were downloaded, 4) Arthropod sequences were excluded and the first 20 homologous sequences were downloaded. Duplicate hits were manually sorted out, and synthetic sequences were removed. For AsFAR, the same sequence set was used as in [21], plus a few recent FAR entries found in GenBank (NCBI). The FAD set was aligned using MUSCLE in MEGA5 [44], and the phylogenetic trees were built using Neighbor-Joining, bootstrap with 1500 replicates, pairwise deletion, and JTT matrix. The FAR set was processed the same way, with the exception that the alignment was performed using ClustalW2 [45]. For a list of all species used in the phylogenies, see Additional file 5.

### Cloning and functional expression of AseΔ11

The Δ11-desaturase-like *A. segetum* ES583599 EST clone, previously described to be 80-fold upregulated in the gland as compared to the body [25], was sequenced using the pTriplEx2 (Clontech) vector-specific primers (VSP) P104 and P105 (primer sequences used in this study are listed in Additional file 6). Sequencing reactions were carried out with the Big Dye Terminator cycle sequencing kit v1.1 (Applied Biosystems), and sequences were analyzed in BioEdit Sequence Alignment Editor [46]. The gene-specific primer (GSP) pFLAseF02D9-s and pFLAseF02D9-as were designed to amplify the full-length ORF with the Advantage® 2 PCR enzyme system (Clontech). The PCR product, AseΔ11, was digested with EcoRI (Promega) and BamHI (Promega), and then cloned using T4 DNA ligase (Invitrogen) at 4°C overnight into the yeast shuttle vector pYEXCHT linearized at the EcoRI and BamHI sites. The presence and directionality of AseΔ11 was verified by sequencing with the vector-specific primers pYEXCHT-F and pYEXCHT-R. Sequenced clones of AseΔ11-pYEXCHT were transformed into the *InvSc1* strain of the yeast *S. cerevisiae* (*MATa his3D1 leu2 trp1-289 ura3-52*, Invitrogen), and were selected at 30°C for URA+/LEU + transformants on SC –ura –leu containing 2% (w/v) glucose. For the assay, individual yeast colonies were inoculated in 5 ml of the same selective liquid medium for 48 h at 30°C and 300 rpm, diluted to an OD_600_ of 0.4 in 250 ml-flasks containing 20 ml selective medium and 2 mM CuSO_4_, and were grown for 48 h at 30°C and 300 rpm. Lipids were extracted from yeast pellets with 500 μl chloroform/methanol (2:1 v:v) at room temperature for 1 h, followed by base-methanolysis as previously described [23] to convert all fatty acids to their corresponding methyl esters. The products were recovered in *n*-hexane prior to GC-MS analysis. The position of double bonds in monoenes was determined by DMDS derivatization [47] followed by GC-MS analysis.

### Cloning and functional expression of AseFAR

FASTA file of the *A. segetum* library contigs were formatted as a BLAST database and searched using a standalone BLAST. Silkworm and European corn borer pgFARs were used as queries in tBLASTn searches to identify the EST contigs with homology to pgFARs. A contig containing a pgFAR-like partial sequence was used as a template for designing a GSP, pFlAseFARas for 5′ RACE PCR using SMARTer RACE cDNA Amplification Kit (Clontech). The resulting 5′-sequence was used to assemble a consensus full-length sequence. A forward GSP pFlAseFARs5 was subsequently designed to amplify the full-length AseFAR in combination with pFlAseFARas using the Advantage® 2 PCR enzyme system (Clontech). The PCR product was then cloned into pYES2.1 (Invitrogen) and all constructs were verified for AseFAR using the vector specific primer (VSP) Gal1 and V5. AseFAR-pYES2.1 was transformed to InvSc1 as described above, and the FAR assay was performed as previously outlined in [21].

### Co-expression of AseΔ11 and AseFAR

The primers pGAL1-vec-F and ptCYC-vec-R were used to add the pYES2.1 *GAL1p* and *CYC1* terminator (*CYC1t*), and a 40 bp pYEXCHT vector homology sequence to AseFAR (Figure 1), by PCR amplification from AseFAR-pYES2.1 with Phusion® High-Fidelity DNA Polymerase (New England Biolabs). One μg of the PCR product was co-transformed with 2 μg PvuII-linearized AseΔ11-pYEXCHT to *InvSc1* yeast using the LiAc/SS carrier DNA/PEG method [48], which promoted homologous recombination and created AseΔ11/*CUP1p*-AseFAR/*GAL1p*-pYEXCHT. To investigate whether the production of Z11-16:OH could be increased by placing the two enzymes and the intermediate substrates into closer proximity, we made an additional construct, which fused the two ORFs of AseΔ11 and AseFAR to a single ORF, hence creating a bifunctional enzyme (Figure 1). The AseΔ11 primers pAs11pyex01s and pAsD11pyex01as, and AseFAR primers pAsFpyex01s and pAsFpYEX01as were used to amplify each corresponding ORF with Phusion®, adding additional homology to both the joining-site (a GC-linker) of the two ORFs and the XhoI-linearized pYEXCHT. The stop codon of AseΔ11 was changed to a proline codon, thus allowing full transcription from the start codon of AseΔ11 to the stop codon of AseFAR (Figure 1). The PCR products and linearized pYEXCHT were co-transformed into *InvSc1* to generate the construct AseΔ11-FAR/*CUP1p*-pYEXCHT. Yeast transformants were selected on SC –ura –leu 2% glucose agar plates, and analyzed by colony PCR for both AseΔ11 and AseFAR using Gal1 and V5 (AseFAR region) or pYEX-F and pYEX-R (AseΔ11 region). Plasmids were rescued into One Shot® TOP10 (Invitrogen) and the constructs were sequenced to confirm the sequence integrity of AseΔ11 and AseFAR with the primers Gal1, V5, pYEXCHT-F, and pYEXCHT-R.

For the small scale assay, yeast transformants were inoculated in 8 mL SC –ura –leu 2% raffinose and incubated over night at 30°C and 300 rpm, followed by dilution to OD_600_ of 0.1 into 8 mL of either the same media (for AseΔ11FAR-pYEXCHT), or SC –ura –leu 2% (w/v) galactose 1% (w/v) tergitol (for AseΔ11-AseFAR-pYEXCHT), 2 mM CuSO_4_, and grown for 24 h at 30°C. The yeast-produced alcohols were extracted from the culture using an equal volume of 8 ml dichloromethane. The solvent was evaporated under a gentle stream of N_2_, and the residue was re-dissolved in 100 μL *n*-hexane containing 10 ng/μL of the external standard (*Z*)-11-tridecanol (Z11-13:OH), prior to analysis by GC-MS. All assays included a pYEXCHT as a negative control. For AseΔ11-AseFAR-pYEXCHT, two positive controls with either AseΔ11-pYEXCHT or AseFAR-pYES2.1 were assayed in parallel. To verify Δ11-activity, the extracted samples of AseΔ11/*CUP1p*-AseFAR/*GAL1p*-pYEXCHT were subjected to base-methanolysis, as described above.

For the large-scale assay, three replicates of AseΔ11/*Cup1p*-AseFAR/*GAL1p*-pYEXCHT transformants were inoculated in 100 ml SC –ura –leu 2% (w/v) raffinose, incubated over night at 30°C and 300 rpm, followed by dilution to OD_600_ of 0.1 in 100 mL SC –ura –leu 2% (w/v) galactose 1% tergitol and 2 mM CuSO_4_ and grown for 48 h at 30°C. The alcohol products were extracted with an equal volume (100 ml) of chloroform:methanol (2:1) and shaken vigorously. The extract was transferred to a round bottom flask to evaporate the solvent using a rotary evaporator, and then re-dissolved in 10 mL dichloromethane including the internal standard Z11-13:OH at 10 ng/μL.

The crude extract of yeast containing ca. 500 μg of total alcohols was purified by thin layer chromatography (TLC) before performing the oxidation. The alcohol fraction was collected in 1 mL dichloromethane in a 4 mL screw-cap vial, and then 10 mg of pyridinium chlorochromate (PCC) was added. The vial was flooded with N_2_ and the mixture was vortexed briefly followed by a 2 h-reaction at room temperature. The reactants were then loaded on a florisil column (0.5 mm i.d.) and eluted with 1 mL of diethyl ether. The collection was evaporated to dryness and re-dissolved in 1 mL hexane before GC quantification and further use.

### GC-MS and GC-EAD

A Hewlett Packard 5890II GC system, coupled to a mass selective detector (HP 5972) and equipped with a medium-polar INNOWax column (100% polyethylene glycol, 30 m × 0.25 mm i.d., 0.25 μm film thickness, Agilent Technologies) was used to analyze all yeast extracts as well as products transformed into aldehydes. The GC–MS was operated in electron impact mode (70 eV), the injector was configured in splitless mode at 220°C, and helium was used as carrier gas (velocity: 30 cm/s). A 50°C oven temperature was maintained for 2 min followed by an increase at a rate of 10°C/min up to 220°C, and held for 20 min. The products were identified by comparison of the retention time and mass spectra with those of the reference compounds. DMDS adducts of fatty acid methyl esters were analyzed with a HP 6890–5975 GC-MS system equipped with a non-polar HP5-MS column (5% (w/v) phenyl-methylpolysiloxane, 30 m × 0.25 mm i.d., 0.25 μm film thickness, Agilent Technologies). The injector was configured in splitless mode and helium was used as carrier gas (velocity: 30 cm/s). The oven temperature was maintained at 80°C for 2 min followed by an increase at a rate of 10 C/min to 180°C, a rate of 3°C/min to 260°C, a final increase to 280°C at a rate of 20°C/min, and then held for 10 min. Products were identified based on characteristic ions according to [47].

GC-EAD was used to identify physiologically active compounds from the oxidized extracts of yeast transformants. Since *A. segetum* mainly uses chain-shortened derivatives that were not present in the co-expression extracts, and therefore would not serve as a good biodetector in this case, we used antennae from *H. virescens* males that respond to Z11-16:Al, as well as other aldehydes [49,50]. An antenna of a three- to five-day-old male was cut at both ends, and mounted to a PRG-2 EAG (10× gain) probe (Syntech, Kirchzarten, Germany) using conductive gel (Blågel, Cefar, Malmö, Sweden). Charcoal-filtered and humidified air passed over the antennal preparation from a glass tube outlet at 5 mm distance from the preparation. The GC effluent to the antenna passed through a heated transfer line set at 230°C.

Samples were analyzed on an Agilent 7890A GC (Agilent Technologies), equipped with a HP-INNOWax column (30 m × 0.25 mm i.d., 0.25 μm film thickness, Agilent Technologies). A split at the outlet of the column allowed a 1:1 division of the GC effluent to the flame ionization detector (FID) and to the antennal preparation. Hydrogen was used as carrier gas at a flow rate of 43 cm/sec and injector temperature was 225°C. The column temperature was maintained at 80°C for 2 min and then increased by 10°C/min to 220°C. Each antennal preparation was used for one or two GC-EAD trials. Data were analyzed with the GC-EAD Pro Version 4.1 software (Syntech, Kirchzarten, Germany).

## Abbreviations

AseΔ11: *Agrotis segetum* Δ11 fatty-acyl-desaturase; AseFAR: *Agrotis segetum* fatty-acyl-reductase; DMDS: Dimethyl disulfide; FAD: Fatty-acyl-desaturase; FAR: Fatty-acyl-reductase; GC-EAD: Gas chromatography with electroantennographic detection; GC-MS: Gas chromatography-mass spectrometry; pgFAR: Pheromone gland fatty-acyl-reductase.

## Competing interests

The authors declare that they have no competing interests.

## Authors’ contributions

ÅKH and CL conceived the project and designed research. ÅKH performed the experiments. ÅKH, TJ, ML, CL and HW analyzed the data. HW and JML contributed reagents/materials/analysis tools. ÅKH wrote the first draft of the manuscript. HW, ML, JML, TJ and CL contributed to the writing of the manuscript. All authors read and approved the final manuscript.

## Supplementary Material

Additional file 1 **GC-MS trace of *Ase Δ*** ***1*** ***1 *** **heterologously expressed in *S. cerevisiae *** **(top) versus yeast negative control (bottom).** Click here for file

Additional file 2 **GC-MS trace of *AseFAR *** **heterologously expressed in *S. cerevisiae *** **supplemented with Z9-14:ME.** Click here for file

Additional file 3 **GC-MS trace of *Ase Δ*** ***1*** ***1-FAR/CUP1p-pYEXCHT *** **(top) versus yeast negative control (bottom).** Click here for file

Additional file 4 **The pheromone biosynthetic pathways of *Agrotis segetum *** **(yellow) and *Heliothis virescens *** **(red) sex pheromone.** Click here for file

Additional file 5 Species abbreviations. Click here for file

Additional file 6 Primer sequences. Click here for file
