# Supplementary figures and images for: A moth pheromone brewery: production of (Z)-11-hexadecenol by heterologous co-expression of two biosynthetic genes from a noctuid moth in a yeast cell factory
Source: Microb Cell Fact. 2013 Dec 13;12:125. doi: 10.1186/1475-2859-12-125 (PMC4126085; doi:10.1186/1475-2859-12-125)

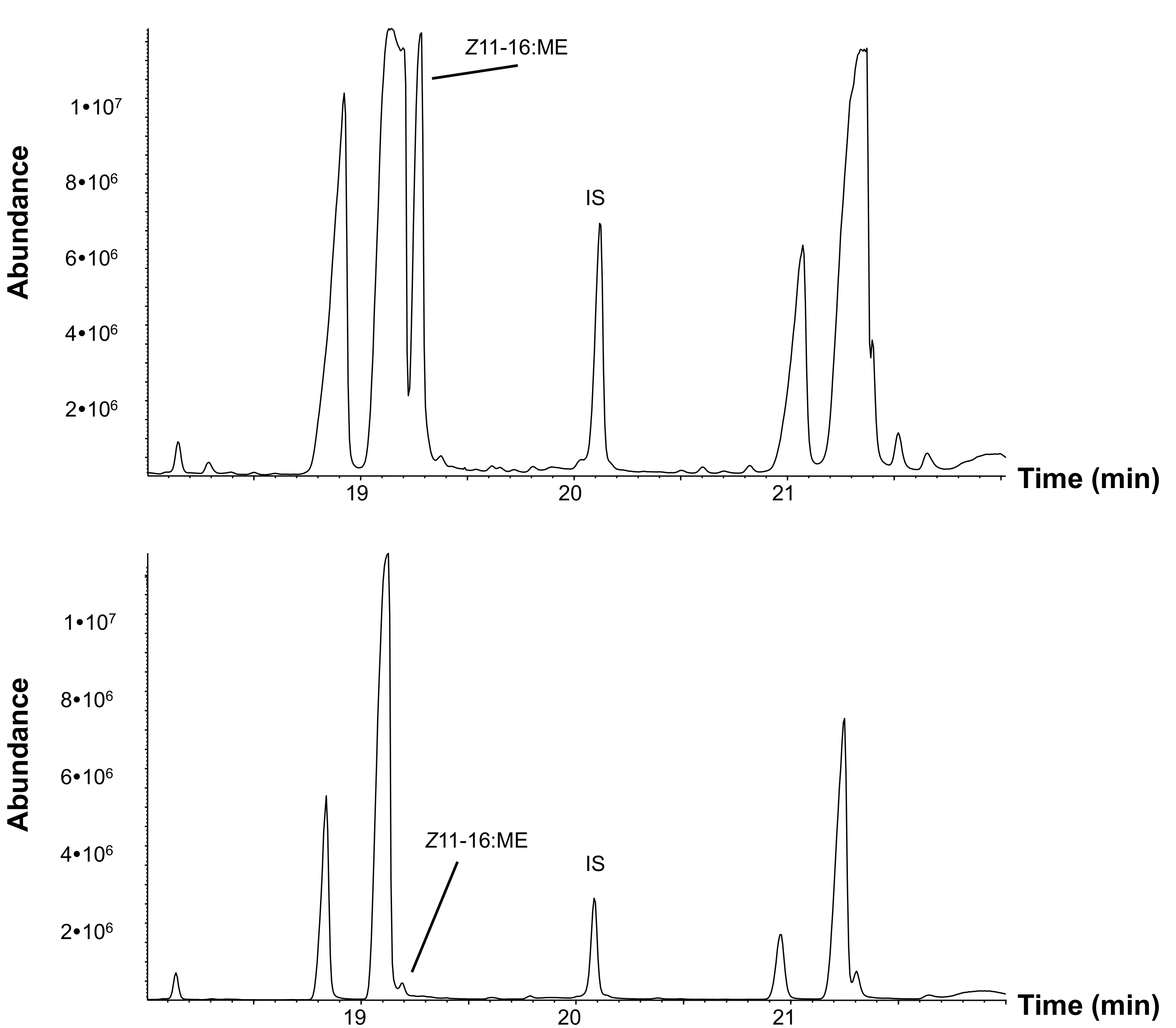

Supplement: Additional file 1 — GC-MS trace of Ase Δ 1 1 heterologously expressed in S. cerevisiae (top) versus yeast negative control (bottom). [file 1475-2859-12-125-S1.tiff]

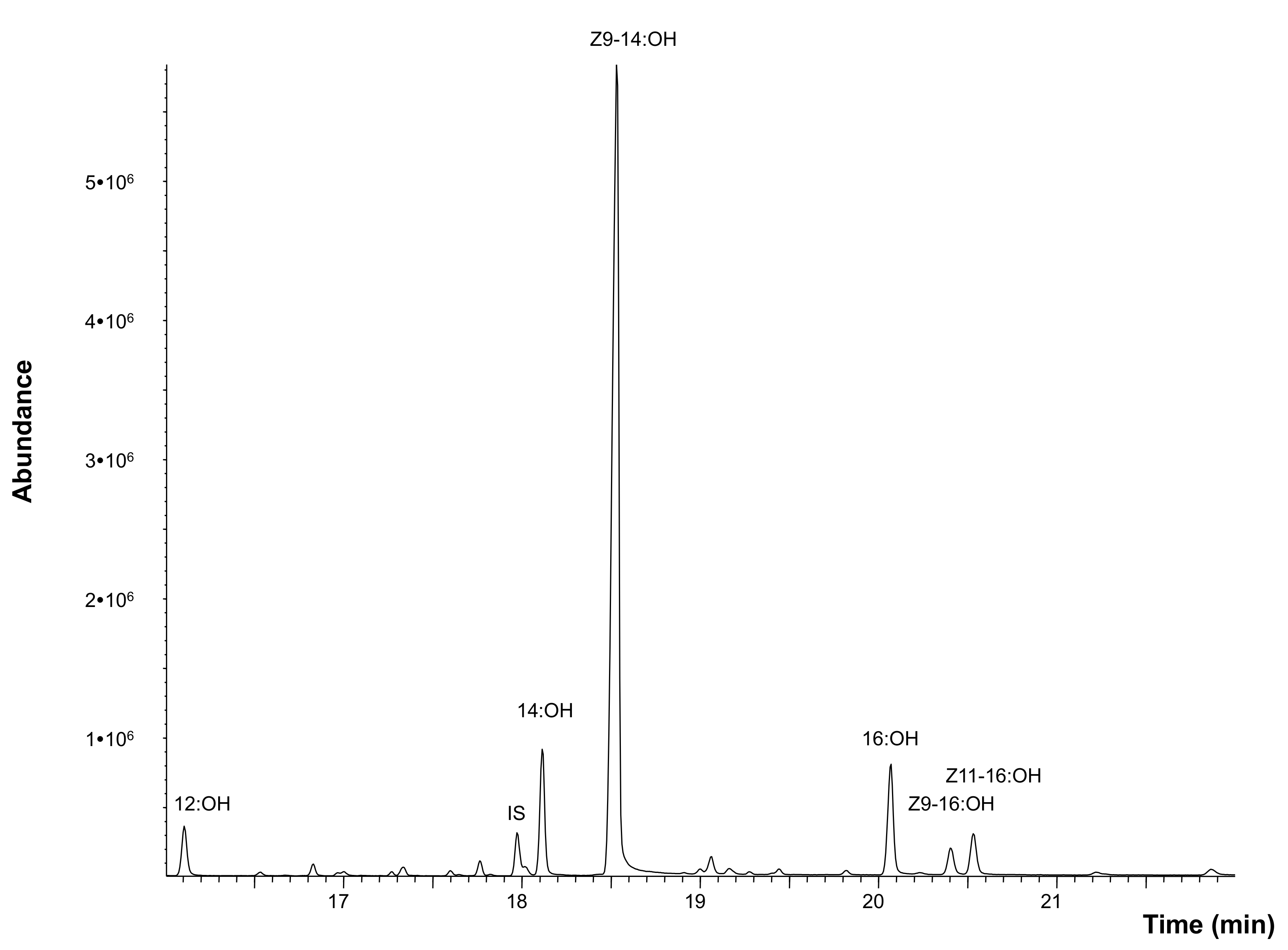

Supplement: Additional file 2 — GC-MS trace of AseFAR heterologously expressed in S. cerevisiae supplemented with Z9-14:ME. [file 1475-2859-12-125-S2.tiff]

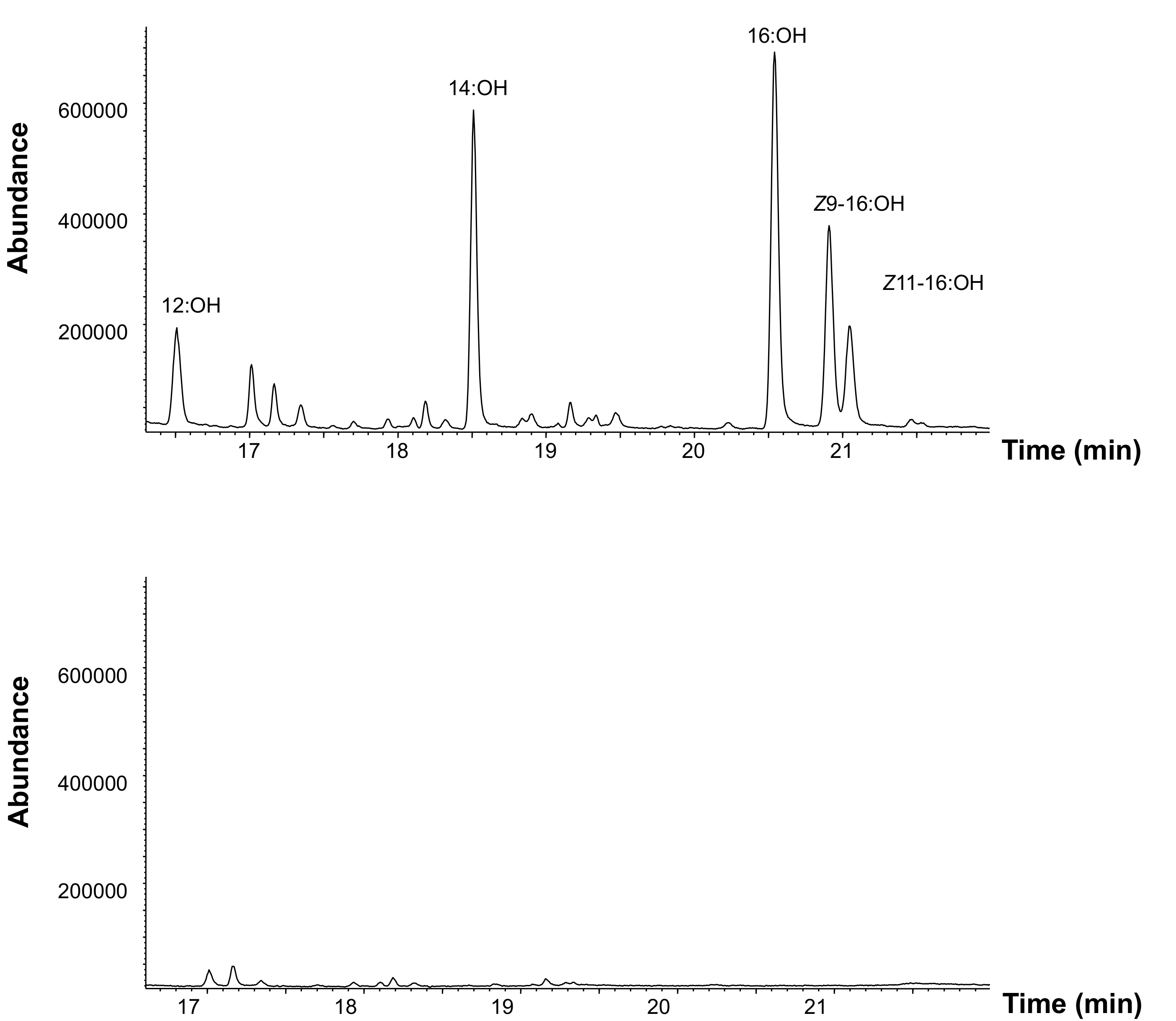

Supplement: Additional file 3 — GC-MS trace of Ase Δ 1 1-FAR/CUP1p-pYEXCHT (top) versus yeast negative control (bottom). [file 1475-2859-12-125-S3.tiff]

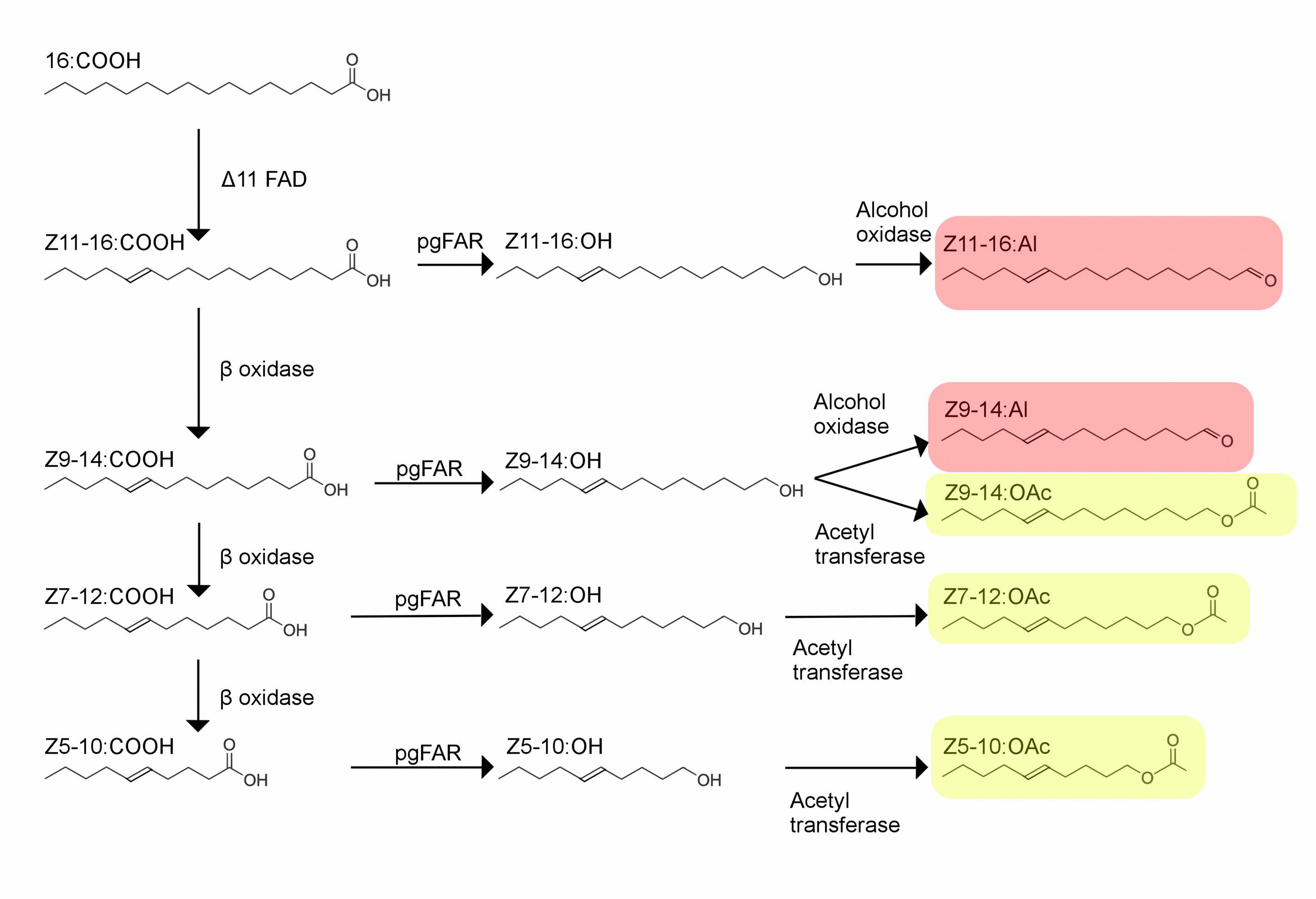

Supplement: Additional file 4 — The pheromone biosynthetic pathways of Agrotis segetum (yellow) and Heliothis virescens (red) sex pheromone. [file 1475-2859-12-125-S4.tiff]
